# The Multifaceted Mas-Related G Protein-Coupled Receptor Member X2 in Allergic Diseases and Beyond

**DOI:** 10.3390/ijms22094421

**Published:** 2021-04-23

**Authors:** Paola Leonor Quan, Marina Sabaté-Brescó, Yanru Guo, Margarita Martín, Gabriel Gastaminza

**Affiliations:** 1 Department of Allergy and Clinical Immunology, Clínica Universidad de Navarra, 31008 Pamplona, Spain; msabate@unav.es (M.S.-B.); gastaminza@unav.es (G.G.); 2 Navarra Health Research Institute (Instituto de Investigación Sanitaria de Navarra) (IdiSNA), 31008 Navarra, Spain; 3 Biochemistry Unit, Biomedicine Department, Faculty of Medicine, University of Barcelona, 08036 Barcelona, Spain; yanruguo@ub.edu (Y.G.); martin_andorra@ub.edu (M.M.); 4 Laboratory of Clinical and Experimental Respiratory Immunoallergy, IDIBAPS, 08036 Barcelona, Spain

**Keywords:** mas-related G protein-coupled receptors, mas-related G protein-coupled receptor member X2 (MRGRPX2), mast cells, hypersensitivity

## Abstract

Recent research on mast cell biology has turned its focus on MRGPRX2, a new member of the Mas-related G protein-coupled subfamily of receptors (Mrgprs), originally described in nociceptive neurons of the dorsal root ganglia. MRGPRX2, a member of this group, is present not only in neurons but also in mast cells (MCs), specifically, and potentially in other cells of the immune system, such as basophils and eosinophils. As emerging new functions for this receptor are studied, a variety of both natural and pharmacologic ligands are being uncovered, linked to the ability to induce receptor-mediated MC activation and degranulation. The diversity of these ligands, characterized in their human, mice, or rat homologues, seems to match that of the receptor’s interactions. Natural ligands include host defense peptides, basic molecules, and key neuropeptides such as substance P and vasointestinal peptide (known for their role in the transmission of pain and itch) as well as eosinophil granule-derived proteins. Exogenous ligands include MC secretagogues such as compound 48/80 and mastoparan, a component of bee wasp venom, and several peptidergic drugs, among which are members of the quinolone family, neuromuscular blocking agents, morphine, and vancomycin. These discoveries shed light on its capacity as a multifaceted participant in naturally occurring responses within immunity and neural stimulus perception, as in responses at the center of immune pathology. In host defense, the mice Mrgprb2 has been proven to aid mast cells in the detection of peptidic molecules from bacteria and in the release of peptides with antimicrobial activities and other immune mediators. There are several potential actions described for it in tissue homeostasis and repair. In the realm of pathologic response, there is evidence to suggest that this receptor is also involved in chronic inflammation. Furthermore, MRGPRX2 has been linked to the pathophysiology of non-IgE-mediated immediate hypersensitivity drug reactions. Different studies have shown its possible role in other allergic diseases as well, such as asthma, atopic dermatitis, contact dermatitis, and chronic spontaneous urticaria. In this review, we sought to cover its function in physiologic processes and responses, as well as in allergic and nonallergic immune disease.

## 1. Introduction

Recent research on mast cell (MC) biology has turned its focus on a newly described subfamily of receptors, Mas-related G protein-coupled receptors (Mrgprs) [1]. These receptors belong to a larger family of receptors, termed G protein-coupled receptors (GPCRs), which contain seven transmembrane domains coupled to heterotrimeric G proteins [2]. Under this subfamily there exists a receptor group termed “X” (MrgprX), which includes four different receptors in humans (X1–X4). Within MrgprX subfamily, a single receptor is expressed primarily in human subjects, as opposed to macaques and rhesus monkeys [3]: the MAS-related G protein-coupled receptor member X2 (MRGPRX2). MRGPRX2 has recently been found to hold important functions in immune response, both physiologic and pathologic, with a universality fit to that of its carrier, the MC. Moreover, it has been described in other key effector cells of type-2 immunity, basophils and eosinophils [4]. In this review, we sought to cover its function in physiologic immune response, as well as in allergic and nonallergic immune diseases, providing an update on the functions identified so far.

## 2. Receptor Family, Structure, and Ligands

GPCRs have been described as ubiquitous proteins that detect distinct and varied physical and chemical stimuli and whose activation launches varied intracellular signaling pathways responsible for key functions within the cell [3]. Within the GPCR family, several receptor subfamilies have yet to be characterized [3]. One of these subfamilies is the Mas-related subfamily, named after its relationship to the Mas gene, a sequence originally isolated from a human epidermoid cell line, and its partner protein, Mas [5,6]. Mas-related GPCRs, or Mrgprs, have slowly come out of the “orphan state” as potential ligands have been discovered. In 2001, Dong et al. first described them as a subfamily of approximately 50 GPCRs related to Mas1 and present in the neurons of the dorsal root ganglia, with varied degrees of expression in different subpopulations of sensory neurons and with potential roles in the regulation of nociceptor function and development and pain [1,2]. Mrgprs have, since then, been recognized as markers for itch-specific lines in the peripheral nervous system, as mediators in functions such as sleep, and as participants in cell functions of proliferation, circulation, and degranulation [3,7,8].

Efforts to describe the structural conformation of the Mrgpr family have been shown to be complex [9], and its three-dimensional architecture is yet to be experimentally predicted. Data on the primary sequence of all Mrgprs show that they lack the main structural elements found in other main class GPCRs, such as rhodopsin [5,9]. More specific and validated molecular predictions of the mrgpr structures are lacking.

**Figure 1:**
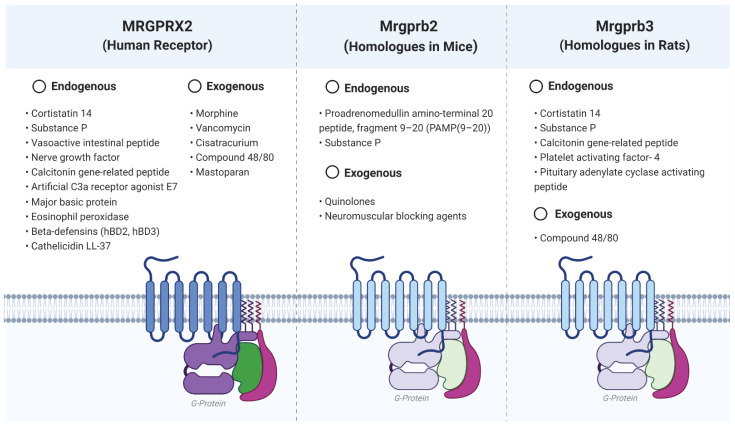
Ligands for the Mas-related G protein-coupled receptor X2 and its functionally convergent homologues in mice and rats. Created using BioRender.com (accessed on 22 April 2021).

Human MRGPRX2 has been found to be expressed in human umbilical cord MCs, together with small amounts of other related receptors, namely, human MRGPRX1 [10,11]. Despite limited knowledge of the receptors’ configuration, the identification of Mrgprb2 and Mrgprb3 as the mouse and rat functionally convergent homologues of human MRGPRX2 have contributed to the practical possibility of new research and specific ligand detection [2,12]. Detected ligands for MRGPRX2 or its homologues are shown in Figure 1. Stimulation of the human MRGPRX2 receptor has been demonstrated by neuropeptides such as substance P (SP) [13,14], nerve growth factor (NGF), calcitonin gene-related peptide (CGRP) and vasoactive intestinal polypeptide (VIP) [13], cortistatin 14 [15], compound 48/80, and, by E7, an artificial C3a receptor agonist [16]. MRGPRX2 has been shown to elicit mast cell degranulation after exposure to human beta-defensins (hBD)2 and hBD3, as well as to cathelicidin LL-37 [11,17]. Other peptides, such as mast cell degranulating peptide, pituitary adenylate cyclase-activating peptide, and platelet-activating factor (PAF)-4, have been shown to induce human and rat MC degranulation through human MRGPRX2 [10]. Protein and enzyme fragments potentially originated from the breakdown products of damaged or dying cells are also capable of MC stimulation through this receptor [18] Tatemoto et al. also showed activation via Mrgprb3 by SP, a ligand whose canonical receptor is the tachykinin receptor neurokinin-1, and a main mediator correlated to the intensity transmission of pain [19] and itch [20]. Furthermore, Green et al. demonstrated that Mrgprb2-silenced mice had reduced mechanical and thermal hypersensitivity and that injection of SP resulted in a decreased influx of CD45+ cells, neutrophils, and monocytes compared to wild-type mice [21]. Eosinophil granule proteins, namely, major basic protein (MBP) and eosinophil peroxidase (EPO), have been proposed as ligands for the receptor [14].

## 3. Mast Cells and Other Cells Expressing MRGPRX2

After the discovery of the Mrgpr family in neurons of the dorsal neural ganglia [1], MRGPRX2 was first described in umbilical cord MCs, making this the second main cell type to be considered a carrier of the receptor family [10,11]. As ubiquitous residents in front-line zones of environmental exposure, MCs are paramount participants and regulators in innate immune responses to pathogens [22,23]. Before complex details of their physiologic role in host response became known, MCs were identified as primary degranulators in allergic disease [24,25], and their receptors and mediators are often the target of biologic treatments for allergic inflammation [26]. To match to their initial physiologic role as first responders, MC architecture is constructed to enhance innate immune activity, namely, a fine-tuned capacity for early pathogen detection [22,27]. As battlefront sentinels, therefore, MCs possess a vast array of membrane receptors geared toward surveillance of pathogens, with a capacity for detecting a variety of danger signals transmitted from tissues and other immune cells, as well as the ability to modulate responses according to the character of the stimulus received [22,26,27]. Receptor presence is known to vary according to the tissue in which a particular MC is present [2].

Specific pathways activated in MCs are not always beneficial, however [22]. Knowledge on the physiologic function of MCs in host response was preceded by the description of its role in allergic reactions [28] mediated by the immunoglobulin receptors. The high-affinity receptor of immunoglobulin E [28], Fc epsilon receptor I (FcƐR1), is the main mediator of allergic response, which holds an original role in immune response to parasites [29], constitutes one of the most well-known initiators of MC degranulation, dependent on the cross-linking of two immunoglobulin E (IgE) molecules with an antigen [30]. Despite the thoroughly established clinical relevance of “IgE-mediated” activation pathways in pathologic responses, alternative activation pathways are being described for MCs with roles in allergic and inflammatory diseases. Receptors for alarmins, such as ST2, an interleukin (IL)-1 family receptor for IL-33, and a receptor for thymic stromal lymphopoietin (TSLP) are involved in MCs’ role in pathologic type-2 inflammation [31]. Stem-cell factor (SCF) and PAF receptors have a role in scaled degranulation during anaphylaxis as well as MC maturation [32]. Within the GPCR realm, members of different subclasses have been shown to mediate MC activity independent of IgE. ADGRE2, a member of the adhesion G protein-coupled receptor subclass, has been shown to induce greater degranulation of transfected LAD2 cells than a control vector [33] Finally, the characterization of the MRGPRX2 receptor, a member of yet another GPCR subclass, has opened novel pathways for research of mast cell biology in the “IgE-independent” realm.

It is unclear whether MRGRPX2 is expressed only in MCs, but evidence suggests the contrary. In 2019, Wedi et al. sought to characterize the functional presence of the receptor in human basophils and eosinophils, and found constitutive expression in both cell types, employing flow cytometry, immunocytochemistry, and immunofluorescence [4]. Furthermore, calcium influx and concentration-dependent degranulation, as well as a promotion of survival induced by anti-MRGPRX2 antibody, suggested a functional role for the receptor in these populations [4]. The fact that MBP and EPO induce histamine release from human skin MCs through MRGPRX2 suggests at least an interaction between the receptor in MCs and eosinophil-produced proteins.

## 4. Activation and Inhibitory Pathways

As MRGPRX2 begins to show a role in pathologic immune response, studies are beginning to gear toward possible inhibitory pathways in its activity within effector cells. Several reports show that activation of this specific receptor increases intracellular calcium [11,34]. Furthermore, a recent study by Occhiuto et al. suggests that store-operated calcium entry via stromal interaction molecule 1 (STIM1), a described mechanism for FcƐR1 activation, may regulate MC activation through MRGPRX2 [34]. Cd300f, an inhibitory receptor shown to regulate IgE-dependent MC reactions, has been shown to inhibit pseudo-allergic responses mediated by Mrgprb2 in mice in vivo, rendering it a possible target in the treatment of MC-induced anaphylaxis [35,36]. Callahan et al. showed a reduction in compound 48/80-induced calcium mobilization (as measured by a change in fluorescence intensity) and degranulation (as measured by a beta-hexosaminidase release assay), after incubation with osthole, in vitro, using the LAD2 MC cell line [37]. Sugammadex, a compound capable of reversing muscle blockade by certain neuromuscular blocking agents (NMBAs), has been shown to reduce mast cell activation induced by some NMBAs via MRGRPRX2 when administered in co-treatment with receptor agonists [38]. This effect was not observed if administration was conducted minutes following stimulation, suggesting a limited role in treatment of acute anaphylaxis. These findings shed light on the emerging possibilities for inhibition of MRGPRX2-induced responses via pharmacologic agents.

## 5. Described Actions of MRGPRX2 in Physiologic Processes and Responses

### 5.1. Host Defense

**Figure 2:**
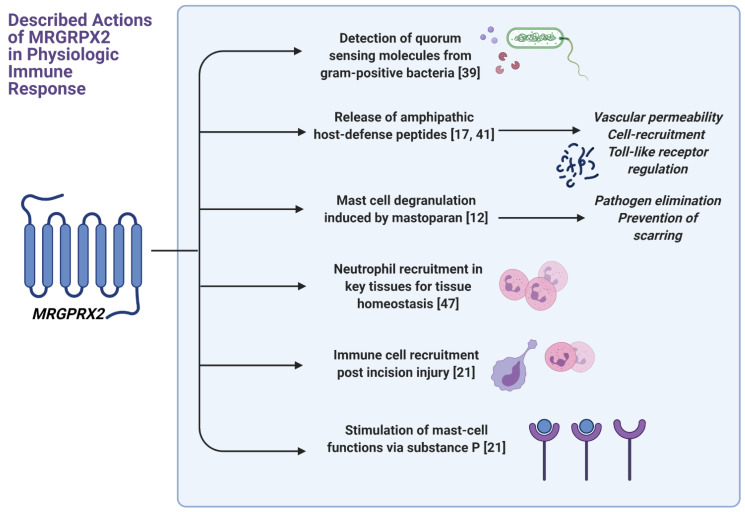
Described actions of the Mas-related G protein-coupled receptor X2 in physiologic immune response. Created using BioRender.com (accessed on 22 April 2021).

With new discoveries in the last few years, it has been proposed that MRGPRX2 may have a key role in the ongoing conversation between innate and adaptative immunity mediated by the mast cell (Figure 2). Its characteristics allow the receptor to be a key player both in threat detection as well as in the direct elimination of pathogens. Pundir et al. recently showed that mast cells recognize several extracellular, autoinducer chemical signals known as quorum-sensing molecules of Gram-positive bacteria through MGRPRX2 [39]. Recent research has demonstrated that MRGPRX2 participates in the stimulation pathways that induce release of amphipathic peptides named host defense peptides (HDPs), as well [40]. HDPs include human β-defensins (hBD), a cathelicidin named LL-37, and lipocalin 2, a peptide with a capacity of blocking Gram-negative bacterial growth [17,41]. The interaction between these molecules is intricate. Subramanian et al. suggested that hBD, namely, hBD2 and hBD3, as well as LL-37, are capable of activating MCs through MRGPRX2 in vitro [11,17,42]. In addition, HDPs have demonstrated the ability to increase Toll-like receptor 4 (TLR-4) expression on MCs, likely enhancing their pathogen-detection properties [43], together with the capacity of increasing vascular permeability or recruiting other immune cells, such as neutrophils, to the infection site [17,44,45]. Peptides such as mastoparan, belonging to wasp venom, induce MC degranulation through this receptor and hold pathogen elimination activity both in the innate and adaptive immunity realms [40,46]. In summary, MRGPRX2 seems to play a relevant role in MC antimicrobial activity induced by peptides.

### 5.2. Tissue Homeostasis and Repair

MCs expressing MRGPRX2 can contribute to tissue homeostasis through immune cell recruitment in key tissues, such as the periodontal area [47]. Mastoparan has been shown to reduce tissue scarring, through MC activation via MRGPRX2/b2. The proposed mechanisms, mediated by MCs, include neutrophil recruitment and potentially the restoration of CD301b+ dendritic cells in the skin, which, in turn, induce renewal of epithelial cells [48]. In postoperative inflammation models, mast cells which do not express Mrgprb2 yield fewer recruitment of CD45+ cells, neutrophils, and monocytes after incision injury [21], suggesting a role in cell recruitment related to tissue disruption, even prior to the presence of pathogen invasion.

### 5.3. Nociception, Inflammatory Pain, and Itch

Presence of the receptor was first described at high levels in small-diameter neurons of the dorsal root ganglia [15], speculating a possible role in nociception [1]. The postoperative inflammation models used by Green et al. showed that both male and female mice lacking Mrgprb2 expression have significant reductions in thermal and mechanical hypersensitivity as well as post-incision swelling [21]. In addition, the activation of MRGPRX2 by neuropeptides further supports a link between the receptor and painful stimulus transmission. McNeil et.al found that SP, a key mediator of pain, which was so far only connected to one receptor, the neurokinin 1 (NK-1R) receptor, is also an agonist of Mrgprb2 and of MRGPRX2, with higher sensitivity to the latter [46]. MRGPRX2, when stimulated by SP, is involved in immune-cell recruitment [21]. Upon stimulation with SP, the human mast cell line LAD2, which express MRGPRX2, releases TNF-α, granulocyte-macrophage colony-stimulating factor (GM-CSF), IL-8, and the chemokines CCL2, CCL3, and CCL4 at a higher rate than LAD2 cells with a knocked-down MRGPRX2 [21]. These findings place MRPGPRX2 at the center of the connection between immune regulation and sensory transmission of neurologic stimuli.

MRGPRX2 is increasingly being found to be involved in itch perception. SP is but one of the known mediators of itch that are ligands for the receptor [2,8,49]. Other than SP, vasoactive intestinal peptide [50], bovine adrenal medulla peptide [15], and compound 48/80 (C48/80) [46] are all known mediators of itch. Furthermore, Reddy et al. found that SP and C48/80 activate native versions of the Mrgpr receptor in mice and do not activate versions with a single amino-acid mutation [49].

### 5.4. Sleep Regulation

A potential physiologic role for MRGPRX2 was first described by Robas et al. in an article directed at characterizing cortistatin-14, a high-affinity ligand for which no specific receptor had been previously described [15]. Cortistatin is known to act mainly through activation of somatostatin receptors [51,52], but also holds functions that suggest interaction with other receptors as well [15]. MRGPRX2 may contribute in the non-somatostatin regulation of sleep patterns through cortistatin, which increases slow-wave sleep without effects on rapid-eye-movement sleep, possibly through the inhibition of pyramidal neurons in at least three regions of the hippocampus, where the receptor has been found to be expressed in moderate levels [15].

### 5.5. Potential Roles of MRGPRX2 in Types of Pathologic Immune Response

**Figure 3:**
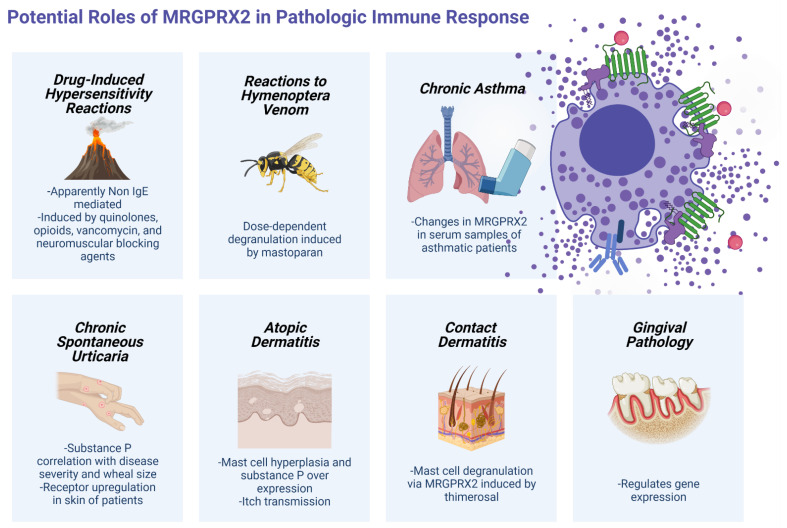
Potential roles of the Mas-related G protein-coupled receptor X2 in pathologic immune response. Created using BioRender.com (accessed on 22 April 2021).

The receptor’s role in the aforementioned physiologic responses makes it a likely target for study in situations of immune dysregulation. Its characteristics may allow us to carefully understand response in non-physiologic conditions that associate MC response. MC have been best characterized in the classic IgE-mediated activation of the high-affinity receptor and its subsequent cascades. There are a series of diseases, however, in which, while mast cell function is confirmed, clinical findings suggest a non-IgE mechanism. Fit to its capacity for interconnection and correlation, the role of MRGPRX2 seems not to be limited to one type of immune pathology, as it seems to be involved in immediate-type hypersensitivity, chronic immunity, and several ambits of the type-2 category of inflammation (Figure 3).

## 6. Immediate-Type Hypersensitivity Reactions

### 6.1. Drug-Induced Hypersensitivity Reactions

The best-characterized pathological function of MCs is in allergy [26,53]. Years of study in drug hypersensitivity have shown that not all immediate-type hypersensitivity reactions are IgE-mediated. The fact that the presence of Mrgprb2 does not impair IgE or other G protein-coupled receptor signaling [46] suggests that the receptor is mainly involved in non-IgE pathways. Furthermore, similarities between peptides that have been found to stimulate MRGPRX2 or its functionally convergent homologue suggested that cationic, peptidergic drugs may stimulate it [46]. This may be particularly true for a certain subset of immediate-type hypersensitivity reactions that seem to be dose-dependent and particularly specific of intravenous administration. Assays conducted by McNeil et al. showed that drugs that contain structural patterns called tetrahydroisoquinolone (THIQ) motifs are capable of activating mast cells through Mrgprb2 very effectively [46].

Such motifs or similar are found to be present in members of the NMBA and fluoroquinolone drug families. Hence, atracurium and members of all NMBA families (except succinylcholine) and ciprofloxacin, as well as icatibant, were found to activate MCs through both receptors, inducing the release of histamine, tumor necrosis factor, prostaglandin D2, and B-hexosaminidase [46].

Knowledge of the role of the receptor on drug reactions has expanded further. Liu et al. found MRGPRX2-induced calcium mobilization and cell degranulation when LAD2 was stimulated with several other fluoroquinolones: fleroxacin, pefloxacin, lomefloxacin, norfloxacin, enoxacin, gatifloxacin, sparfloxacin, levofloxacin, and moxifloxacin [54]. In addition, Hamamura-Yasuno et al. replicated these findings in HEK293 cells transfected with human or dog MRGPRX2, using ciprofloxacin, levofloxacin, gatifloxacin, and pazufloxacin [55]. These findings were connected to the mechanisms of pseudo-allergic reactions by showing the presence of clinical signs of inflammation in rats treated with fluoroquinolones whose MCs carried Mrgprb3 [54]. Following these previously described findings, our team conducted a comprehensive analysis of the mast cell response mediated by the receptor in drugs used in perioperative procedures and anesthesia [56]. These drug groups meet the criteria of intravenous administration, at sufficient concentrations to stimulate the receptor, as well as belonging to groups that contain susceptible motifs. Our study found that MC degranulation, as analyzed by beta-hexosaminidase release and CD63 expression, was reduced in cells with a silenced MRGPRX2, after stimulation by cisatracurium (confirming findings on neuromuscular blocking agents), morphine, and vancomycin, whereas remifentanil, teicoplanin, amoxicillin, and diclofenac did not seem to induce degranulation via the receptor [56]. Furthermore, sera from patients who experienced anaphylactoid reactions during anesthesia induced degranulation at a lower degree when MRGPRX2 was knocked down [56]. The drugs found to activate MCs via the receptor were capable of triggering hypersensitivity reactions during the first administration, resulted in doubtful or unexpected results in skin testing, or were considered non-specific histamine releasers [56]. These findings confirmed the role of the receptor in pseudo-allergic and non-allergic reactions induced by drugs. The type of granule-release response observed in mast cells when MRGPRX2 is stimulated, for example, by SP [57], matches the clinical characteristics of immediate reactions observed with these drugs, which seem to be dose-dependent and of a shorter duration than pure IgE-mediated reactions [56]. MRGPRX2 activation induces production of small, spherical granules, whereas FcƐRI activation induces the production of heterogeneous granules that are longer-lasting, more fit to the sustained anaphylactic responses observed in purely IgE-mediated disease, such as food allergy [57,58].

The range of drugs known to induce MRGPRX2/b2-dependent MC activation is, at the moment, expanding [59,60]. In mouse models of passive cutaneous anaphylaxis, hindpaw inflammation induced by Iopamidol, an iodinated contrast, was not only found to be associated with mast cells, but also to be MrgprB2-dependent [61].

The capacity for receptor-induced activation by peptidic drugs does not yet explain the fact that some individuals suffer pseudo-allergic reactions with symptoms compatible with immediate hypersensitivity, which do not always show positivity during skin testing reactions, and others do not. Individual receptor levels [62], as well as the functionality of the receptor, which can be slightly altered by changes in the gene sequence or post-translational modifications, may be the key to understanding the distinct responses of certain individuals as compared to others. Mutations in MRGPRX2 have been shown to prevent the interaction between known agonists, such as SP and compound 48/80 [63]. Variants of the MRGPRX2 protein occurring due to missense mutations have been found to show absence of response when stimulated by known stimulants, namely, SP, hemokinin-1, β-defensin-3, and a drug ligand, icatibant [64]. The findings of Reddy et al. regarding the effect of single amino-acid changes in the induction of itch through the receptor [49] support a potential role of genetic polymorphisms in relation to variable drug-induced reactions. Some studies have begun to assess the impact of MRGPRX2 variants, observed in patients with drug reactions, on activity of the receptor [65], with no enhanced degranulation observed so far. The specific role of different mutations on the clinical epidemiology of pseudo-allergic responses to drugs has yet to be studied further.

### 6.2. Reactions to Hymenoptera Venom

Mastoparan, a cationic peptide component of bee wasp venom, induces dose-dependent degranulation in MC lines, specifically in MRGPRX2-containing lineages [48]. This finding may suggest a possible involvement of this receptor in the physiologic repair and bacterial defense process after stings and possibly in the pathologic immediate inflammatory responses observed in certain subjects after venom exposure.

## 7. Prolonged Type-2 Inflammation

MC activation is not only closely linked to immediate hypersensitivity. The role of MCs in chronic inflammation is also well established [24].

### 7.1. Chronic Asthma

MCs have been described as known amplifiers of IL-33-mediated immune response in chronic asthma [23]. New studies have pointed to MRGPRX2 in this respiratory disease. An et al. assessed MRGPRX2 levels in serum from healthy and asthmatic patients (further divided into allergic asthmatic and non-allergic asthmatic patients) [66]. The mean serum MRGPRX2 level as measured by ELISA was found to be significantly higher in the asthma group as compared to the control group. Nevertheless, current data do not support the expression of MRGPRX2 in the lung and, thus, further research is needed to clarify the meaning of such findings [24,62]. Finally, receptor presence and activity may be involved not only in the presence and control of disease but also in acute exacerbations, such as those induced by viral agents [2].

### 7.2. Skin and Connective Tissue Pathologies

Expression of MRGPRX2 has been found to be tissue dependent. Mrgprb2, for example, is highly specific to connective tissue mast cells [46]. In humans, MRGPRX2 is also predominantly expressed in connective tissue MC, especially from skin and adipose tissue [67], and skin has been also described as the tissue with the highest expression based on RNA-seq data [63]. Other than cutaneous signs of immediate inflammation found in drug hypersensitivity, the receptor is likely to have a role in some chronic skin disorders. 

Several studies have addressed the impact of regulatory factors in MRGPRX2 biology [32]. Data show that MCs treated with retinoic acid, a component capable of potentiating inflammatory cytokines in mast cells [68], decreased MRGPRX2 gene expression and resulted in a lower amount of histamine released when triggered with C48/80 [32], a finding observed by others in mice models, as well [69]. Babina et al. also found that the presence of SCF, mainly, as well as the additive presence of interleukin-4, strengthened the FcƐRI activation pathway while down-regulating the MRGPRX2 one [62,70]. In other studies, however, acute MC exposure to bursts of IL-33 secretion enhanced both activation routes as well as degranulation, while chronic exposure to IL-33 reduced their activity, especially that of the MRGPRX2 pathway [32]. Finally, TSLP, which can be secreted by epithelial cells and fibroblasts and is a driver of type-2 immunity, has been shown to enhance MRGPRX2-mediated MC degranulation. This suggests an intricate feedback loop-type behavior that holds complex interrelations involving the main players in chronic skin disease.

Wang et al. proposed a possible role for the receptor in atopic dermatitis, for instance [32]. Their paper argued that MCs’ participation in the pathology of atopic dermatitis is likely mediated by a pathway other than the allergic FcƐRI-IgE pathway, that a change in the relationship between MCs and neuronal units must be involved, and that specific bacterial components may activate MRGPRX2 directly [32]. Presence of MC hyperplasia and over expression of neuropeptides such as SP [71] and tryptase may suggest the presence of an intrinsic, cyclical relationship between nerves, in which neuropeptides activate MCs and MCs release neuropeptides that influence neurons, with MRGPRX2 at its center [32]. This also allows for a partial explanation of the important role of bacterial over-infection in atopic dermatitis pathology. Presence of bacterial elements in the skin may induce MC activation through the receptor, as discussed earlier [32,41]. A deeper study of the chronicity and degree of activation would help further characterize this role. Finally, the fact that single amino-acid alterations in MRGPRX2 induce changes in the transmission of itch [49] may partially explain the genetic predisposition of the disease.

A new connection is being discovered between MRGPRX2 and contact dermatitis. Peng et al. discovered that thimerosal, a preservative known for inducing contact dermatitis, is capable of inducing mast cell degranulation and increase of calcium concentration in HEK293 cells transfected with either MRGPRX2 or mrgprb2, and that LAD2 cells with a downregulated receptor showed reduced degranulation when exposed to the agent [72]. Presence of thimerosal induces contact dermatitis in dorsal skin and footpad swelling of mice with a normal receptor, an effect that is attenuated when the receptor is knocked out [72]. Interestingly, the receptor seems to mediate both an immediate, non-IgE reaction as well as a delayed hypersensitivity-type reaction in mice, evidence that the receptor has multiple capabilities in distinct types of hypersensitivities. Furthermore, the pseudo-allergic reactions induced by thimerosal have been shows to be dose-dependent, both in terms of presence of edema and vasodilation [72], a factor that sheds light on the receptor’s role in certain drug hypersensitivity reactions, which seem to remain under control when infusion rate is changed, as with vancomycin [56].

The role of MRGPRX2 in skin disease may spread to other types of chronic immune pathologies. Chronic spontaneous urticaria (CSU) is characterized by the occurrence of widespread daily or nearly daily itchy wheals for a period of at least six weeks, often accompanied with angioedema. Recently, Fujisawa et al. demonstrated that skin MC of patients diagnosed with CSU expressed the MRGPRX2 receptor at higher levels than in control individuals [14]. In their study, Fujisawa et al. found that SP causes degranulation in skin-derived cultured MC, and these responses were reduced when the MRGPRX2 gene was silenced [14]. SP is, as mentioned before, a well-known role player in the physiology of itch, a likely independent inducer of histamine release, and a stimulant for basophil degranulation in CSU patients [73]. Furthermore, serum SP levels [73], as well as serum MRGPRX2 levels [74], are correlated with disease severity. Intradermal administration of SP induces larger wheal size in patients with idiopathic CSU when compared to healthy individuals, without an increase in MC number or histamine content, suggesting a functional, not quantity-related alteration in MC biology [4,14]. A functional defect or alteration could be associated to, among other findings, the presence or difference in expression of receptors that lead to activation. The reasons for upregulation of MRGPRX2 in the skin of CSU patients is not yet explained, but an augmented expression of the receptor may make these patients’ skin more sensitive to stimulation by SP and other neuropeptides, contributing to its pathogenesis. 

Recently, other MRGPRX2 ligands, such as rocuronium, have been shown to cause increased skin reactivity in CSU patients [75]. In other types of urticaria, such as vibratory urticaria, for example, other G protein-coupled mast cell receptors have induced MC activation through mechanical vibration [23]. The role of MC receptors that are independent of known IgE mechanisms, such as MRGPRX2, may very well explain symptoms and treatment response in at least some CSU patients. Furthermore, MRGPRX2 has been found to be expressed in basophils and eosinophils, with the capacity to cause cell degranulation upon stimulation with anti-MRGPRX2 [4]. The receptor is also capable of recruiting eosinophils, which coexist with MC in the lesions of these patients, produce proteins such as MBP and eosinophil peroxidase (EPO), and induce histamine liberation in MC, an effect that is absent when MRGPRX2 is inactive [14].

A new role was recently proposed by Chompunud et al. for the receptor in gingival pathology. The number of MCs that express MRGPRX2 was increased in gingiva of patients with chronic periodontitis [76]. Chompunud proposed that, as expression of HDPs is upregulated in patients with periodontitis, mass production of inflammatory mediators induced by HDPs may have a role in the pathogenesis of periodontal disease [40].

## 8. Pending Aspects and New Directions for Study

There is much room to expand studies in this receptor’s role in immune pathology and allergy. Focused studies are needed to further characterize its functions in a variety of contexts. In addition, understanding of the receptor’s role in varying pathologies opens a door for new therapeutic strategies. Furthermore, some compounds have been shown to inhibit MRGPRX2-mediated MC activation, in vivo and in vitro [77,78]. Clinical application of these findings needs still to be explored.

There is still much to clear up on the genetics of the receptor. MRGPRX2 genetic variants have been shown to influence receptor-activating capacity, for instance, by altering ligand binding properties or intracellular signaling functions [40,63,79]. To date, more than 200 variants (excluding synonymous variants) have been reported (GnomeAD v2.1.1, June 2020) most of them with frequencies lower than 0.1% and not appearing in homozygosis. A complete description of the genetics of the receptor will open possibilities, not only for detection of the relationships between mutations and susceptibility for pseudo-allergic reactions with drugs, but also for a role in the genetic predisposition of atopic individuals or individuals with chronic autoimmune disease.

Studies oriented toward the interaction and collaboration with other receptors in physiologic and pathologic response may help clarify the role of the receptor as a main mediator of immune pathology.

As a main mediator in immediate hypersensitivity reactions, Weller affirmed that it is of critical importance to define the role of the MRGPRX2 pathway in anaphylactoid reactions [80]. Other necessary aspects to study include the characterization of the dose-dependent relationship between the administration of endovenous medication and induction of anaphylaxis.

Furthermore, the specific role of MRGPRX2 in the sensitivity and specificity of skin testing for drug hypersensitivity needs to be defined. According to the response published for Kelso et al. [81], a new understanding of skin testing in light of the existence of the MRGPRX2 pathway must substitute our previous one. The sensitivity of skin testing when suspecting IgE-mediated allergy exists at dosing thresholds that are considered “non-irritative” according to current standardization. It is possible that sensitivity for “MRGPRX2-induced” reactions exists at a different threshold, a dose-dependent threshold that we usually avoid in order to rule out “irritative” skin testing [81], and which may very well be inducing localized reactions that are non-IgE but that are indicative of this receptor’s activity. It is important to conduct a study that correlates the existence of MRGPRX2-type reaction and includes the dosing threshold considered as “irritative” as part of the testing spectrum. In drug hypersensitivity, the receptor could represent a pathway by means of which to conduct in vitro diagnosis. A study on the correlation between in vivo skin testing studies and in vivo studies is still missing.

The role of the receptor in anaphylaxis and the presence of MC in mast cell activation syndromes, as well as reported cases of both quinolone and hymenoptera venom-induced reactions [82], has led to the speculation that it may hold a central role in these syndromes [80,82]. Further studies are required on the description of the quantitative and functional presence of MRGPRX2 in mastocytosis and mast cell activation syndromes with a link to clinical activity.

The world of MRGPRX2 holds enormous potential for the study and understanding of even more physiologic and immune pathology. Our role is to continue expanding our knowledge of its importance in regulated and dysregulated processes. Important possibilities lie in characterizing a receptor that may serve as a future target for biologic therapy, for in vitro diagnosis, and for a new spectrum of clinical disease.

## Data Availability

Not applicable.
